# Supplementary material for: Role of Sirtuins in the Pathobiology of Onco-Hematological Diseases: A PROSPERO-Registered Study and In Silico Analysis
Source: Cancers (Basel). 2022 Sep 23;14(19):4611. doi: 10.3390/cancers14194611 (PMC9561980; doi:10.3390/cancers14194611)
Supplement: Supplementary file 1 [file cancers-14-04611-s001.zip › cancers-1779669-supplementary.pdf]

Role of Sirtuins in the Pathobiology of Onco-Hematological Diseases: A PROSPERO-Registered Study and In Silico Analysis

João Vitor Caetano Goes, Luiz Gustavo Carvalho, Roberta Taiane Germano de Oliveira, Mayara Magna de Lima Melo, Lázaro Antônio Campanha Novaes, Daniel Antunes Moreno, Paola Gyuliane Gonçalves, Carlos Victor Montefusco-Pereira, Ronald Feitosa Pinheiro and Howard Lopes Ribeiro Junior

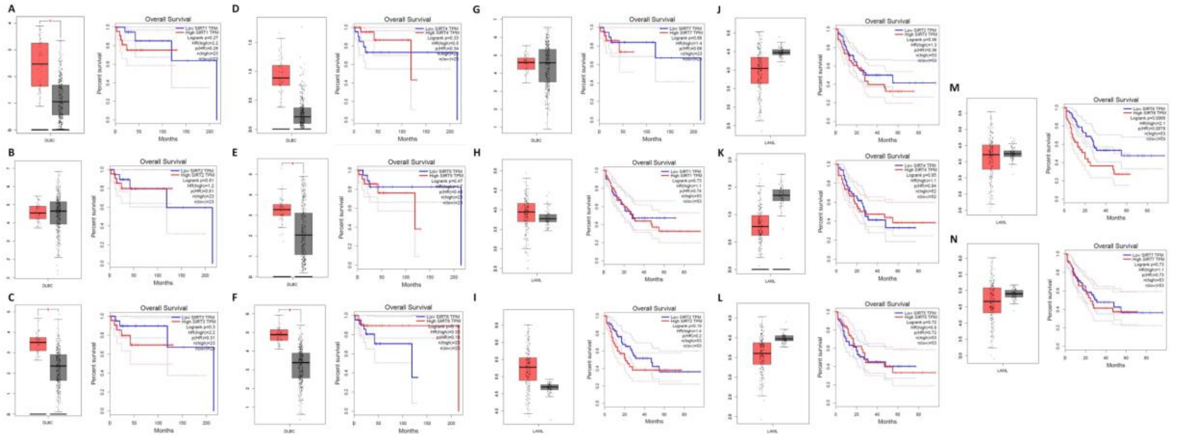

Figure S1. Differential Sirtuins gene expression and overall survival analysis in AML and DBLC diseases (tumor samples × normal samples). Plots **A** to **G** refer to the analysis of differential gene expression (plot on the left; normal versus tumor) and survival (plot on the right) for DLBC. Plots **H** to **N** refer to the analysis of differential gene expression (plot on the left; normal versus tumor) and survival (plot on the right) for AML.
